# Innate Biomineralization

**DOI:** 10.3390/ijms21144820

**Published:** 2020-07-08

**Authors:** Erming Tian, Fumiya Watanabe, Betty Martin, Maurizio Zangari

**Affiliations:** 1 Department of Hematology Oncology, University of Arkansas for Medical Sciences, Little Rock, AR 72205, USA; tianerming@uams.edu; 2 The Center for Integrative Nanotechnology Sciences, University of Arkansas at Little Rock, Little Rock, AR 72204, USA; fxwatanabe@ualr.edu; 3 Arkansas Nano and Bio Materials Characterization Facility, University of Arkansas, Fayetteville, AR 72701, USA; emartin@uark.edu

**Keywords:** biomineralization, hydroxyapatite, alkaline phosphatase, glycerophosphate, crystallinity, nanomaterial

## Abstract

In vertebrates, biomineralization is a feature considered unique to mature osteoblasts and odontoblasts by which they synthesize hydroxyapatite (HAP), which is deposited in the collagen matrix to construct endoskeleton. For many decades, the mechanisms that modulate differentiation and maturation of these specialized cells have been sought as a key to understanding bone-remodeling defects. Here, we report that biomineralization is an innate ability of all mammalian cells, irrespective of cell type or maturation stage. This innate biomineralization is triggered by the concomitant exposure of living cells to three indispensable elements: calcium ion, phosphoester salt, and alkaline phosphatase. Any given somatic cell, including undifferentiated mononuclear cells, can undergo a biomineralization process to produce calcium-phosphate agglomerates. The biologically generated minerals under such conditions are composed of genuine HAP crystallites of Ca_10_(PO_4_)_6_(OH)_2_ and 5–10 nanometer (nm) in size. This discovery will profoundly improve our understanding of bone metabolism and ectopic calcifications.

## 1. Introduction

Bone remodeling is tightly regulated through a wide variety of signaling pathways [1,2] that couple bone resorption by osteoclasts [3,4] and bone formation by osteoblasts [5,6,7,8,9,10], and imbalances in these mechanisms result in bone diseases [11]. It has been assumed that only differentiated, mature osteogenic cells could produce hydroxyapatite (HAP), which is deposited in a collagen matrix to harden bones in the process of biomineralization [12,13]. Fundamental questions about this assumption were raised, however, by the recent discovery of controlled HAP biomineralization in ~810 million-year-old fossils of primitive eukaryotes [14]. The earliest known vertebrate came into existence 300 million years later [15], so the function of HAP in these early unicellular organisms is not clear. In present-day invertebrates, which lack an internal skeleton, HAP formation has been observed on the mandibular teeth of most crustaceans, suggesting that biomineralization is widely conserved across Kingdom Animalia [16,17]. Moreover, inappropriate biomineralization of soft tissues (e.g., cardiovascular tissue) is defined as ectopic calcification [18,19,20]. In previous studies, the induction of osteoblast differentiation was considered the essential first step of biomineralization [21,22,23]. By morphology, osteoblasts are almost indistinguishable from fibroblasts. By gene expression profiles, osteoblasts are similar to fibroblasts. Moreover, no evidence indicates that biomineralization is orchestrated by specific genes expressed in osteoblasts [5,24,25]. In this context, osteoblasts are viewed as sophisticated fibroblasts, which can be merely identified by measuring a mineralized extracellular matrix when the cells are exposed to an environment containing calcium ions, β-glycerophosphate, ascorbic acid, dexamethasone, and serum (fetal bovine serum, FBS) for a period of 3–4 weeks [26,27]. We can now demonstrate that calcium ion, an acyclic alkane (C_n_H_2n+2_) phosphoester salt, and alkaline phosphatase are three indispensable elements governing the biomineralization in any given somatic cell, regardless of type, origin, and maturity.

## 2. Results

### 2.1. The Essential Elements for Biomineralization

**Figure 1:**
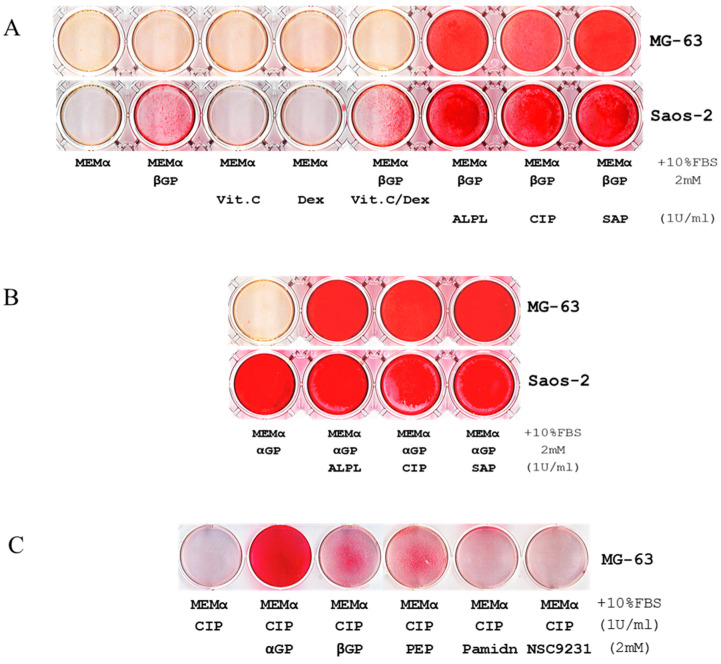

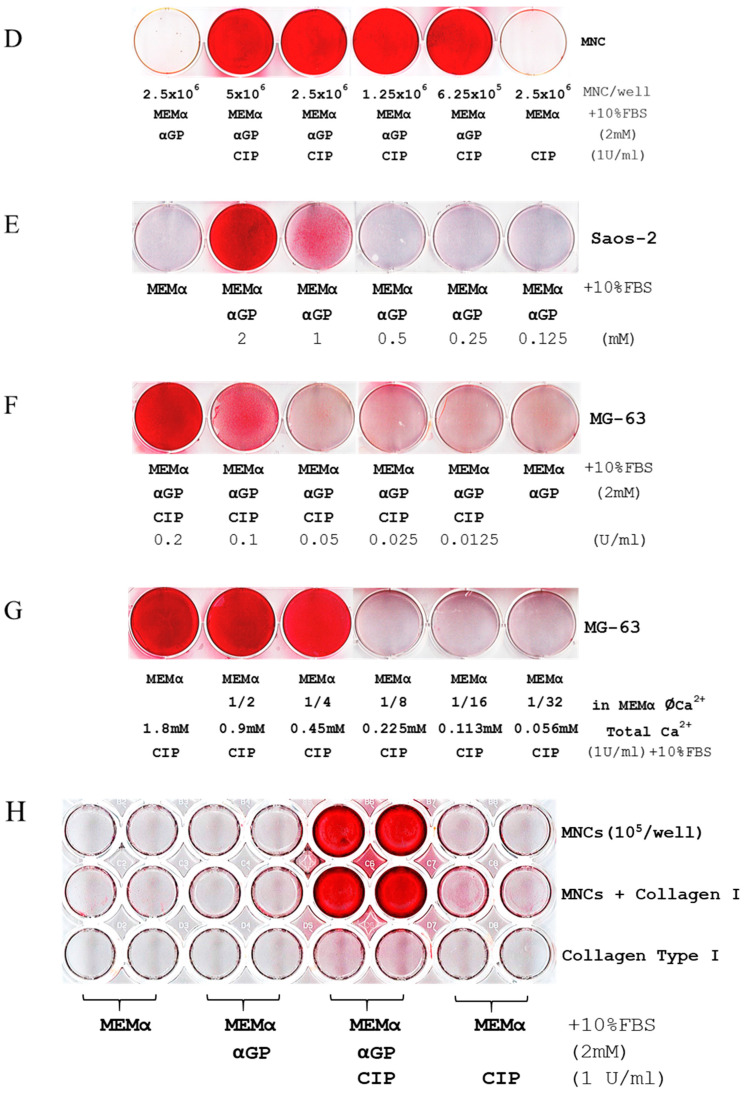
Calcium ion (Ca^2+^), a phosphoester salt, and alkaline phosphatase (ALP) are essential for promoting biomineralization in MG-63 and Saos-2 cell lines and human blood mononuclear cells (MNCs). (**A**) Alizarin Red S assays (ARS) show mineralization within 7 days, visualized as the intensity of red staining, of Saos-2 cells in MEMα/10% FBS supplemented only with βGP or the combination of βGP/ascorbic acid (Vit. C)/dexamethasone (Dex); MG-63 cell line was inactive under similar conditions. No mineralization was observed in either cell line cultured with MEMα/10% FBS supplemented with Vit. C or Dex. In MEMα/10% FBS supplemented with βGP and ALPL, calf intestinal ALP (CIP), or shrimp ALP (SAP), MG-63 and Saos-2 cell lines were mineralized. (**B**) Similar results were iterated when αGP was used instead of βGP. (**C**) Compared to βGP and phosphoenolpyruvate monosodium (PEP), αGP was the most efficient phosphoester salt elicited biomineralization. Pamidronate (Pamidn) and glycerophosphoric acid (NSC9231) did not elicit the reaction. (**D**) Human MNCs also have an innate ability of mineralization in 7 days without the induction of cellular differentiation (top row indicates initial cell counts per well in a 6-well plate). (**E**–**G**) Titration assays indicated that biomineralization depended on the doses of αGP, CIP, and Ca^2+^. (**H**) In a 48-well plate, human blood MNCs were seeded (10^5^/well) and exposed to MEMα/10% FBS, the medium supplemented with αGP, with αGP and CIP, or with CIP for 7 days (the media were changed on day 4). On day 7, ARS indicates that biomineralization occurred in MNCs exposed to MEMα/10% FBS supplemented with αGP and CIP in the uncoated wells (top row) and the wells coated with Collagen Type I, rat tail (middle row). Biomineralization did not occur if αGP or CIP was missing. None of the cell-less wells coated with Collagen Type I was positive for HAP by ARS (bottom row). Each of the tests was in duplicates.

We used Alizarin Red S (ARS) staining assays to investigate biomineralization in two human osteosarcoma cell lines, Saos-2 and MG-63. Under different culture conditions, we observed that Saos-2 cell line could mineralize within seven days of culture in minimum essential medium alpha with 10% FBS (MEMα/10% FBS) supplemented only with disodium β-glycerophosphate (βGP), but MG-63 could not (Figure 1A). Similar results were observed after 21-28 days of incubations (images are not shown). Gene expression profiling and western blot analysis determined that Saos-2 cells expressed high levels of tissue-nonspecific alkaline phosphatase (ALPL) (Figure S1). To explore the role of ALPL in biomineralization, recombinant human ALPL and βGP were added to MG-63 and Saos-2 cell lines cultured in MEMα/10% FBS; within seven days, biomineralization was observed in both cell lines (Figure 1A), suggesting that such reaction requires concomitant presences of ALPL and βGP in MEMα, which contains 1.8 mM calcium. Notably, similar results reiterated when human ALPL was substituted by alkaline phosphatase from calf intestine (CIP) or from shrimp hepatopancreas (SAP) (Figure 1A) and when βGP was substituted with either disodium α-glycerophosphate (αGP) or sodium phospho(enol)pyruvate (PEP) (Figure 1B,C). When bisphosphonate (pamidronate) or glycerophosphoric acid (NSC 9231) was supplemented as an organic phosphate source, biomineralization did not occur (Figure 1C). Further, biomineralization was performed well without ascorbic acid (Vit. C), suggesting that it may be unnecessary or even deleterious to the process (Figure 1A and Figure S2). The titration assays indicated that biomineralization was dose-dependent on αGP, ALP, and calcium (Figure 1E–G), and the reaction did not occur if any one of the three elements was missing (Figure 1 and Figures S2 and S3).

### 2.2. Biomineralization Is an Innate Ability of Any Given Mammalian Cells

In addition to MG-63 and Saos-2 cell lines, we investigated biomineralization in other mammalian cells under similar conditions and without inducing differentiation. These included 26 human cell lines derived from osteoblast, bone marrow stroma, embryo, muscle, breast cancer, colon cancer, prostate cancer, cervical carcinoma, leukemia, lymphoma, multiple myeloma (Figure S3A), undifferentiated human mononuclear cells (MNCs) from peripheral blood (Figure 1D); and two mouse cell lines (Figure S3B). Within seven days, ARS demonstrated that a wide variety of human and mouse cells have the innate ability of self-mineralization. Without living cells, biomineralization reaction did not occur in the cell-less wells that were coated with Collagen Type I (rat tail) and containing MEMα/10% FBS, αGP, and ALP after seven-day incubation (Figure 1H).

### 2.3. The Pathway of Biomineralization

**Figure 2:**
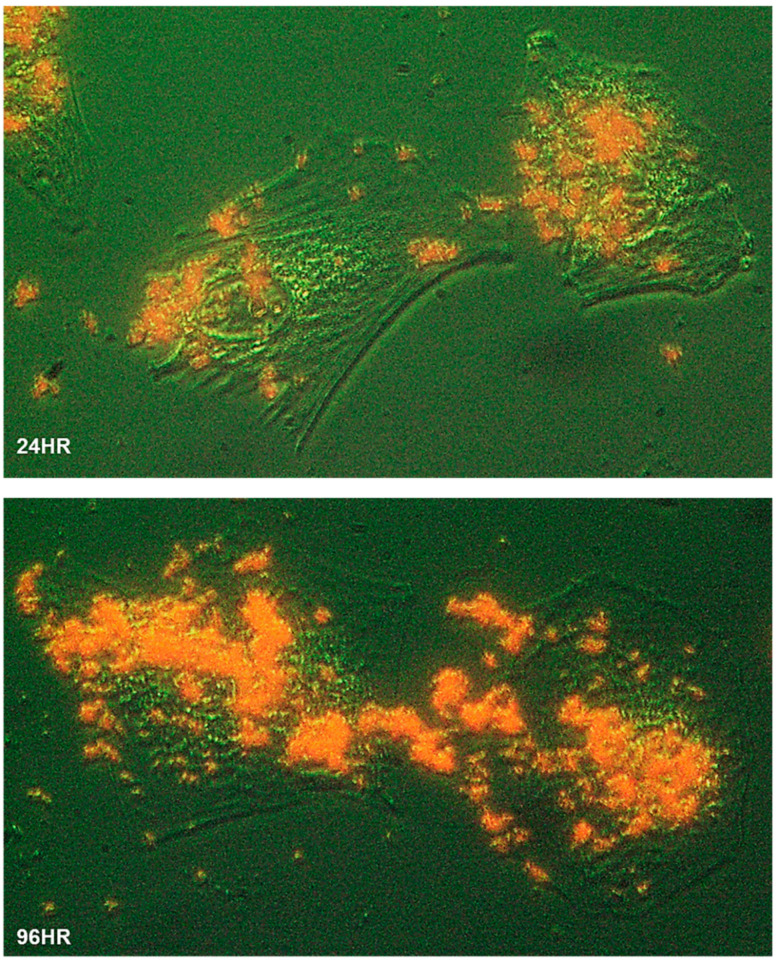
Biomineralization of human cells. The Saos-2 cells grew at low confluency on a glass slide immersed in MEMα/10% FBS with αGP (2 mM) for 24 h (24HR) and 96 h (96HR), respectively. The slides were washed, fixed, and stained with Alizarin Red S. The cell morphology was illustrated using optical phase-contrast (with pseudo-green background), and HAP minerals were in red under a ZEISS inverted microscope equipped with an Infinity 3 digital camera and imaging software. Cells were magnified 400×.

**Figure 3:**
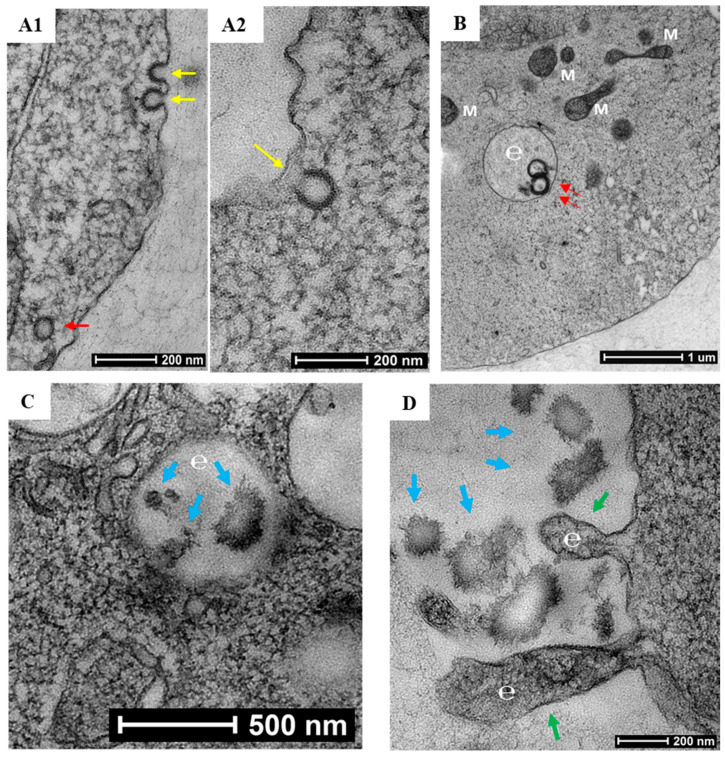
Electron micrographs of biomineralization. K562 cells were cultured for 72 h in MEMα containing 10% FBS, αGP (2 mM), and CIP (1 U/mL). (**A1–2**) Formation of caveolae (yellow arrows) initiated endocytosis of mineral matrix at the cytoplasmic membrane. (**B**,**C**) Caveolar endocytosis (red arrows) transported the mineral matrixes to the endosomes (e), where calcium phosphate agglomerates were synthesized (blue arrows; M = mitochondrion). (**D**) Endosomes (e) budded from the cell membrane (green arrows) to release the mineral agglomerates (blue arrows) into the extracellular space. Bars indicate various scales within the images.

To characterize the process of biomineralization, we monitored the mineral formation in Saos-2 cells cultured in MEMα/10% FBS supplemented with 2 mM of αGP. ARS staining demonstrated that biomineralization occurred at cytoplasmic membrane and cytosol of the adherent cells within 24 h of incubation. A large amount of intracellular accumulation and extracellular secretion of HAP were observed after 96 h of incubation (Figure 2). Further, we investigated this biological process using high-resolution electron microscopy. A nonadherent leukemia cell line, K562, was cultured for 72 h in MEMα/10% FBS supplemented with αGP and CIP. In preparation for transmission electron microscopy (TEM), cell morphology was preserved with high-pressure freezing and freeze-substitution [28]. Serial sections of the cell ultrastructure revealed that the formation of mineral caveolae at the cytoplasmic membrane was the first step of biomineralization (Figure 3A1,A2). Endocytosis of caveolae transported the mineral matrixes into endosomes (Figure 3B), where the calcium phosphate agglomerates were constructed (Figure 3C). Eventually, the mineral agglomerates were carried to the membrane by endosomes and then released into the extracellular space (Figure 3D).

### 2.4. Crystallinity and Atomic Composition of the Mineral Agglomerates

**Figure 4:**
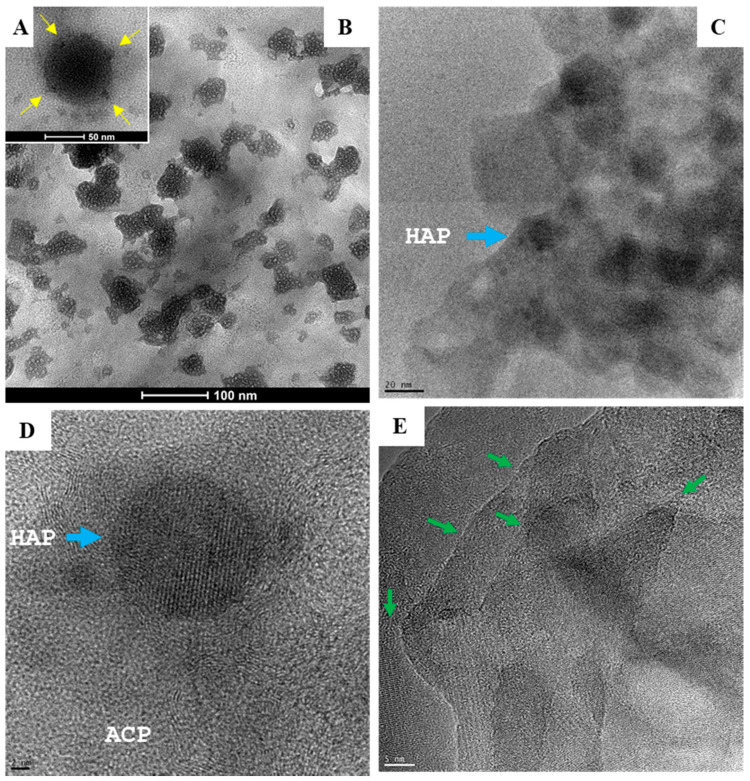
Electron micrographs depict two types of amorphous calcium phosphate (ACP) precursors. (**A**) Large spherical particles (50–100 nm) with remarkable electron-dense areas (inset, yellow arrows) are typical of chemically synthetized nanoparticles (scale bar = 50 nm). (**B**–**D**) Biologically synthetized agglomerates of much smaller granules composed of hydroxyapatite (HAP) crystallites (5–10 nm; blue arrow) produced by human cells grown for 7 days in MEMα/10% FBS with αGP (2 mM) and CIP (1 U/mL). Scale bars: (**B**) = 100 nm; (**C**) = 20 nm; (**D**) = 2 nm. (**E**) Boundaries (green arrows) formed between HAP grains (scale bar = 5 nm).

**Figure 5:**
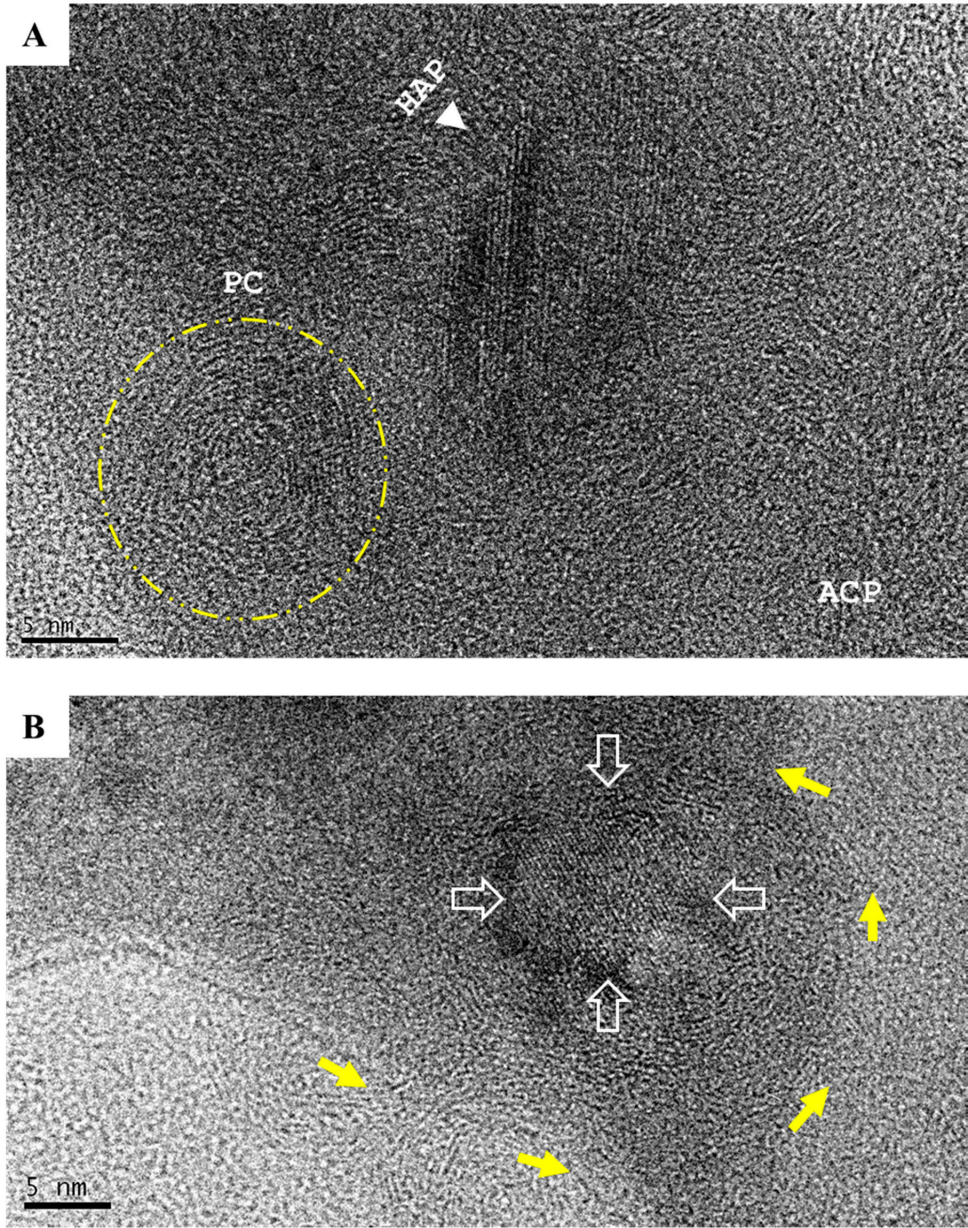

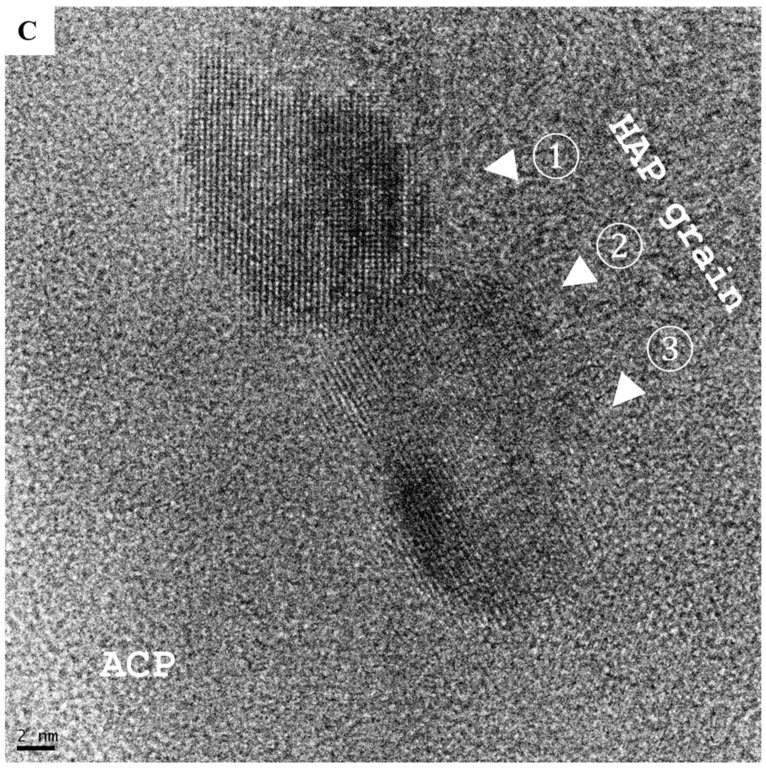
The development of hydroxyapatite (HAP). (**A**) Amorphous calcium phosphate (ACP) coiling (yellow arrows) was a remarkable precrystalline stage that formed polycrystalline masses (PC, in yellow circle). (**B**) Primary crystallization events occurred at the center of the coiling polycrystalline mass (yellow arrows) and resulted in 5–10 nm focus with the crystallographic texture of HAP (hollow arrows; scale bar = 5 nm). (**C**) Primary crystallization triggered a chain reaction that expanded HAP to crystallite grains (white arrows and numbers discrete HAP crystallites; scale bar = 2 nm).

Biologically generated mineral agglomerates from Saos-2, MG-63, MCF-7, PC-3, K562, and RPMI8226 cell lines and MNCs were purified and examined under high-resolution TEM. Unlike chemically synthetized large (>50 nm) spherical particles ([9] and (Figure 4A)), the agglomerates from biomineralization were composed of small granules containing stochastic amorphous calcium phosphate (ACP) (Figure 4B–D), which was ultimately transformed into crystalline HAP. Before crystallization, ACP coiling occurred as a precrystalline stage that aggregated ACP into a polycrystalline mass (“onion ring” in Figure 5A). The primary transformation occurred at the center of coiled ACP with an explicit crystallographic texture of HAP in sizes of 5–10 nm (Figure 5B). This primary event triggered a chain reaction that expanded HAP to crystallite grains (Figure 5C). The formation of grain boundaries (Figure 4E) indicated that the agglomerates could further disintegrate into thin films of HAP grains, with an approximate thickness of 5–10 nm fit into the spaces (~40 nm) between collagen fibrils in bone [28,29].

**Figure 6:**
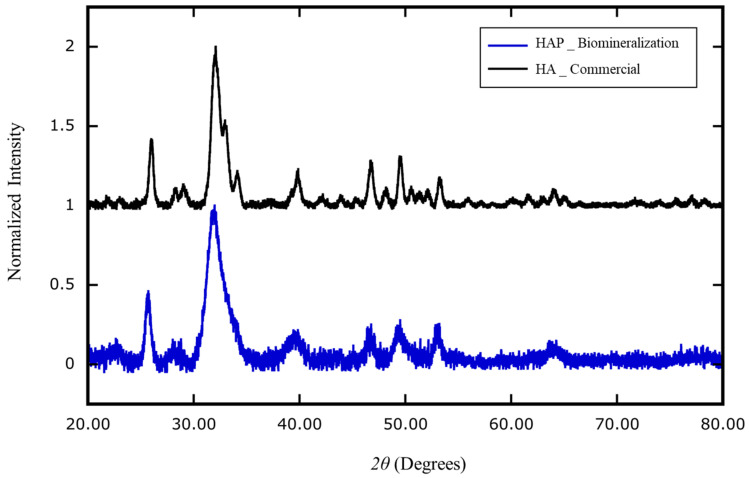
X-ray diffraction analysis of the composition of nanoparticles generated by cell biomineralization versus industrial production. The mineral extracted from human blood mononuclear cells (HAP _ Biomineralization) cultured in MEMα/10% FBS supplemented with αGP (2 mM) and CIP (1 U/mL) for 7 days was identified as homogeneous hydroxyapatite (HAP) (Ca_10_(PO_4_)_6_(OH)_2_, blue). The industrial product of recovered bony material was identified as heterogeneous calcium-phosphate hydrate (Ca_3_(PO_4_)_2_∙xH_2_O) (HA _ Commercial; black).

The composition and crystallinity of the biologically generated minerals were analyzed with an X-ray diffractometer [30,31]. The atomic composition of the extracted nanocrystals was genuine HAP, Ca_10_(PO_4_)_6_(OH)_2_; the atomic composition of the commercial bony nanoparticles was identified as calcium phosphate hydrates, Ca_3_(PO_4_)_2_∙xH_2_O (Figure 6).

## 3. Discussion

Our experiments indicate that ALP from hierarchically distant species (human, bovine, and shrimp) can function as isozymes (Figure 1, Figures S2 and S3), despite substantial differences in their primary structures (Figure S4). This signifies that the controlled HAP biomineralization, which was observed in fossils of unicellular eukaryotes that are ~810 million years old [14] and in extant invertebrates [15,16], is the result of an innate ability of Eukarya that underlies their evolution and survival.

Phosphorus is a key element of bone and a core component of buffer systems that maintain pH homeostasis in the body. Based on our current results, αGP is one of the most efficient acyclic alkane (C_n_H_2n+2_) phosphoester salts for promoting biomineralization (Figure 1 and Figures S2 and S3). In eukaryotes, αGP is an intermediate metabolite of lipid metabolism that contributes to the mitochondrial electron transport chain [32]. In prokaryotes, which lack mitochondria, lipid metabolism occurs in the cytosol to release αGP to the extracellular space [33,34]. In bacteria–animal symbioses, the host eukaryotic cells can “outsource” the αGP produced by prokaryotes to sustain their biological activities [35,36].

The human genome contains four ALP genes: intestinal alkaline phosphatase (ALPI), germ-cell alkaline phosphatase (ALPG), placental alkaline phosphatase (ALPP), and ALPL. ALPI, ALPG, and ALPP are generally inactive (Figure S1). ALPL is present at high levels in bone, liver, kidney, brain, skin, and vascular endothelial cells [37], which are typical locations of cancer metastasis and sites of ectopic calcification. Although ALPs function primarily to catalyze the hydrolysis of phosphoric ester from organic compounds known as dephosphorylation under basic pH conditions, the precise functions of these isozymes at more acidic and physiological conditions are poorly understood. A century ago, Robert Robison, PhD (1883–1941), discovered that a phosphoric esterase (i.e., ALP) is essential for bone mineralization [38,39,40]. Our study repeatedly demonstrated that innate biomineralization could not be achieved when mammalian cells exposed to MEMα comprised of Ca^2+^, inorganic phosphate (1.01 M of NaH_2_PO_4_), and FBS within seven days, even by adding βGP or αGP; unless an ALP was also present (Figure 1 and Figures S2 and S3). Hence, our study has elaborated the crucial function of ALP in control of biomineralization.

## 4. Materials and Methods

### 4.1. Cell Lines

The following mammalian cell lines were obtained from the American Type Culture Collection: human osteoblast hFOB1.19; human osteosarcoma MG-63 and Saos-2; human bone marrow stroma HS-5; human embryonic kidney HEK-293; human rhabdomyosarcoma RD; human mammary gland adenocarcinoma MDA-MB-231 and MCF-7; human colorectal adenocarcinoma SW480, SW620, COLO 205, and COLO 32DM; human prostate carcinoma DU145, LNCaP FGC, and PC-3; human cervical adenocarcinoma HeLa; human leukemia HL-60, K562, and THP1; human lymphoma U937; human myeloma plasma cells HCI-H929, OPM2, RPMI8226, and U266; mouse fibroblast NIH/3T3; and mouse myoblast C2C12. Human myeloma cell lines ARK, ARP1, and CAG were developed in-house. The JJN3 myeloma cell line was provided by Michael Kuehl, MD (National Cancer Institute, Bethesda, MD, USA). Human peripheral blood mononuclear cells were obtained from ZENBIO (Research Triangle Park, NC, USA). All cell lines were cultured in minimum essential medium alpha containing 10% fetal bovine serum (MEMα/10% FBS), penicillin/streptomycin (50 µg/mL of each), and L-glutamine (2 mM).

### 4.2. Materials

The following were purchased from ThermoFisher Scientific (Carlsbad, CA, USA): MEMα with or without phenol red; MEMα with or without ascorbic acid (vitamin C); MEMα with or without calcium (Ca^2+^); penicillin/streptomycin; L-glutamine; TaqMan gene expression assays for ALPG (Hs00741068_g1), ALPI (Hs00357579_g1), ALPL (Hs10129144_m1), ALPP (Hs00740632_gH), GAPDH (Hs99999905_m1); universal PCR master mix. Recombinant human ALPL was purchased from R&D Systems (Minneapolis, MN, USA). Rabbit anti-human ALPL antibody, calf intestinal ALP (CIP, ≥10 DEA U/mg), disodium β-glycerophosphate (βGP), phospho(enol)pyruvate monosodium (PEP), pamidronate, dexamethasone, and Alizarin Red S solution were purchased from Sigma-Aldrich (St. Louis, MO, USA). CIP (≥10,000 U/mL), shrimp hepatopancreas ALP (≥1000 unit/mL), and p-nitrophenyl phosphate kits were purchased from New England BioLabs (Ipswich, MA, USA). FBS was purchased from Atlanta Biologicals (Flowery Branch, GA, USA). Disodium α-glycerophosphate hydrate (1 M of αGP; Glycophos) was purchased from Fresenius Kabi (Lake Zurich, IL, USA), and glycerophosphoric acid (NSC 9231) was obtained from the Developmental Therapeutics Program (NCI, USA). Collagen Type I, rat tail, stock solution was obtained from BD Biosciences Discovery Labware (Waltham, MA, USA).

### 4.3. Quantitative RT-PCR and Western Immunoblot Detection

Total RNA was extracted from cells with a RNeasy Plus mini kit (Qiagen, Hilden, Germany). Reverse transcription was carried out with 500 ng of total RNA with the SuperScript III first-strand system with random hexamer primers, according to the manufacturer’s instructions (ThermoFisher Scientific). cDNA derived from 10 ng of total RNA was used for gene expression assays in TaqMan real-time PCR (20 μL reaction mix); TaqMan assays ran 40 thermocycles for amplification. Quantitative expression of ALPG, ALPI, ALPL, or ALPP gene was calculated based on ΔΔCT relative to GAPDH expression. For protein electrophoresis, each lane was loaded with 20 μg of cell lysate in RIPA lysis buffer (Santa Cruz Biotechnology, Dallas, TX, USA). After electro-blotting to PVDF membrane, a WesternBreeze kit was used for immunodetection with antibodies to human ALPL and GAPDH (Santa Cruz Biotechnology, CA, USA).

### 4.4. Biomineralization Assays and Alizarin Red S Staining

All human and mouse cell lines and human peripheral blood mononuclear cells (MNCs) were maintained in MEMα/10% FBS with penicillin/streptomycin and glutamine in a 37 °C humidified incubator with 5% CO2. In biomineralization assays, the concentrations of αGP (or βGP, NSC 9132, pamidronate, or PEP) as the primary source of organic phosphorus was standardized at 2 mM; and ALPL, CIP, or SAP as a primary source of ALP was standardized at 1 unit/mL (U/mL), respectively. Ascorbic acid (vitamin C) was added at 50 µg/mL (0.284 mM). Dexamethasone (Dex) was added at 100 nM. For adherent cell lines, the assays were performed after 70% confluence was reached in 24-, 12-, or 6-well plates (ThermoFisher Scientific). For nonadherent cell lines, assays were performed in triplicate at an initial density of 1 × 10^4^ cell/well in 96-well plates (ThermoFisher Scientific). The medium was replaced after 4 days. Alizarin Red S staining was performed at room temperature by removing the culture medium, washing the cells twice with 1× PBS (pH 7.4), fixing the cells in 10% neutral buffered formalin (Richard-Allan Scientific, Kalamazoo, MI, USA) for 30 min, rinsing twice with Milli-Q water, staining with Alizarin Red S for 30 min, and destaining twice in Milli-Q water. A ZEISS inverted fluorescence/brightfield microscope equipped with an Infinity 3 digital camera and software system was used for imaging.

### 4.5. Coating Cell Culture Plate with Collagen Type I, Rat Tail for Mineralization Assays

To coat a 48-well plate with Collagen Type I at 5 µg/cm^2^, the stock solution was diluted to 25 µg/mL in 17.5 mM of acetic acid and aliquoted 0.8 mL into each well. After incubating at room temperature for one hour, the Collagen Type I solution was removed. Each well was rinsed with 1xPBS (pH 7.4) and air-dried at room temperature. Human blood MNCs cells were suspended in the conditioned media and distributed into each well (1 × 10^5^ cell/well) proceeded for 7-day incubation. The media were changed on day 4.

### 4.6. Hydroxyapatite Purification for X-Ray Diffraction and Electron Microscopy

The precipitated minerals were collected by scraping, washed twice with 1× PBS (pH 7.4), and suspended in sodium hydroxide (10%) for 30 min. Mineral content was extracted twice with acetone or chloroform and washed twice with 100% ethanol. For X-ray diffraction, purified minerals were air-dried in a spin-vacuum for 30 min at 60 °C. The crystallinity, size, texture, and homogeneity of the dry powder were analyzed with a Bruker D8-Discover X-Ray Diffractometer. For high-resolution transmission electron microscopy, the mineral suspension (in 100% ethanol) was ground manually in a glass grinder and dropped onto a carbon-coated copper grid (Sigma–Aldrich) to allow the ethanol to evaporate. The ultrastructure was examined under a transmission electron microscope (FEI Tecnai F20 200 keV, JEM-2100F, or FEI Titan 80-3000) equipped with a field emission gun (0.1-nm lattice resolution).

## 5. Conclusions

Our study clearly indicates that biomineralization is an innate ability of any given somatic cell and requires the concomitant presence of three indispensable elements—Ca^2+^, a phosphoester salt, and an ALP isozyme. We now propose that bone regeneration and ectopic calcification are governed by the local balance of these three factors.

## 6. Patents

A provisional intellectual property protection has been filed to the U.S. Patent and Trademark Office on 10-16-2019 (OMB 0651-0032).

## Supplementary Materials

Supplementary materials can be found at https://www.mdpi.com/1422-0067/21/14/4820/s1.

## Abbreviations

HAP	Hydroxyapatite
Ca^2+^	Calcium
ARS	Alizarin Red S
ALP	Alkaline phosphatase
TEM	Transmission electron microscopy
XRD	X-ray diffraction
