# Supplementary material for: Innate Biomineralization
Source: Int J Mol Sci. 2020 Jul 8;21(14):4820. doi: 10.3390/ijms21144820 (PMC7404118; doi:10.3390/ijms21144820)
Supplement: Supplementary file 1 [file ijms-21-04820-s001.pdf]

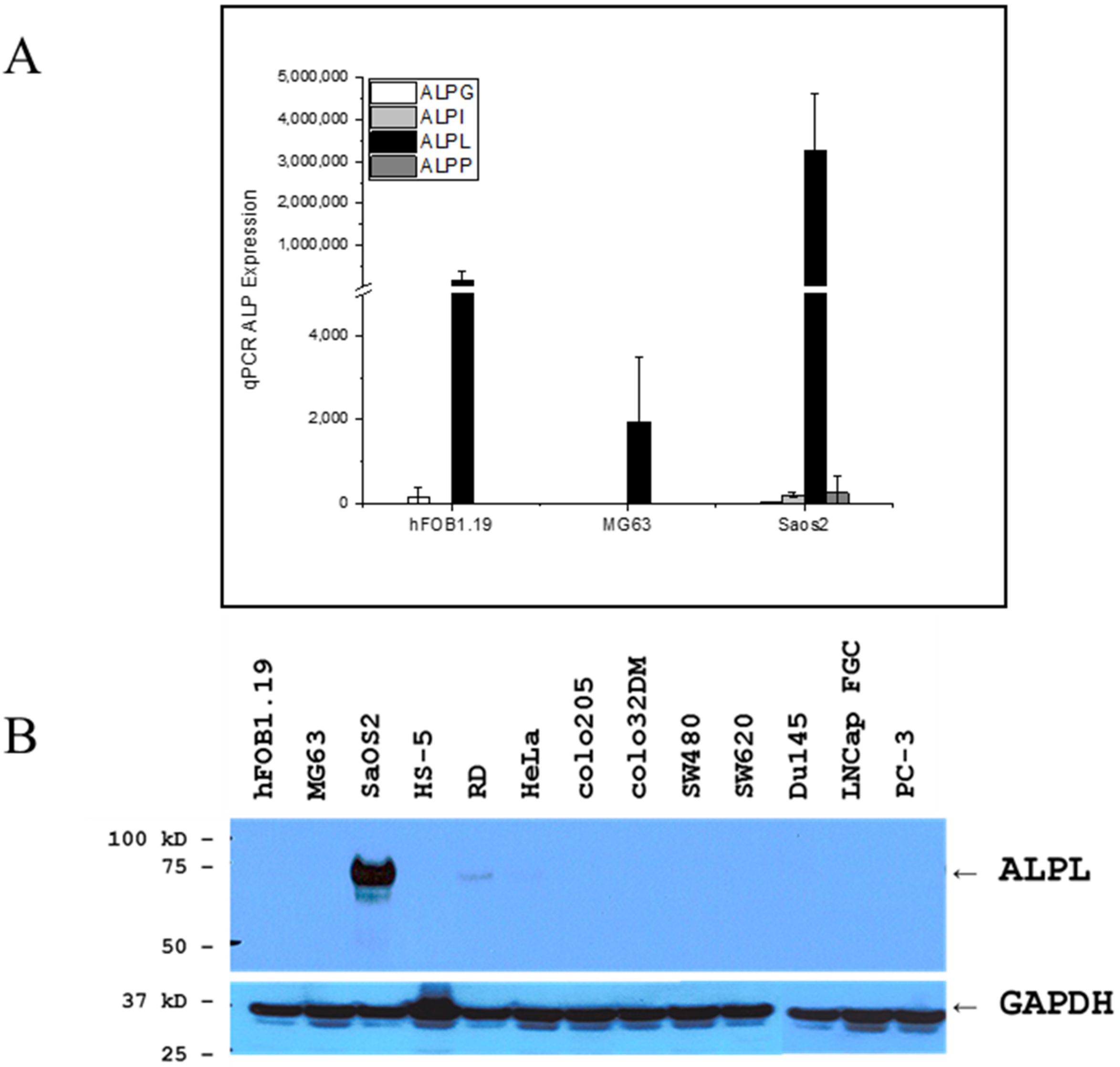

Figure. S1. Gene expression profiling and western blot detection of human alkaline phosphatase (ALP) in cell lines. (A) TaqMan quantitative RT-PCR of human ALPs (ALPG, ALPI, ALPL, and ALPP) in hFOB1.19, MG63, and Saos-2 cell lines. Error bars indicate the standard deviations of three individual RT-PCR assays in triplicate. (B) Western blot of ALPL and GAPDH in 13 human cell lines (20 μg of total protein from cell lysate per lane).

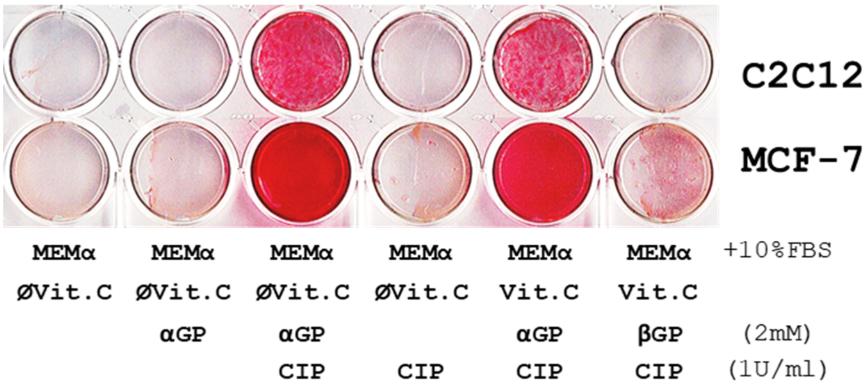

Figure. S2. Biomineralization of C2C12 and MCF-7 cells was dependent on *α*GP (2 mM) and CIP (1 U/ml) supplemented MEM*α*/10% FBS with ascorbic acid (Vit. C) or without ascorbic acid (ØVit. C). The cells were cultured for 7 days (the media were changed on day 4) and then stained with Alizarin Red S.

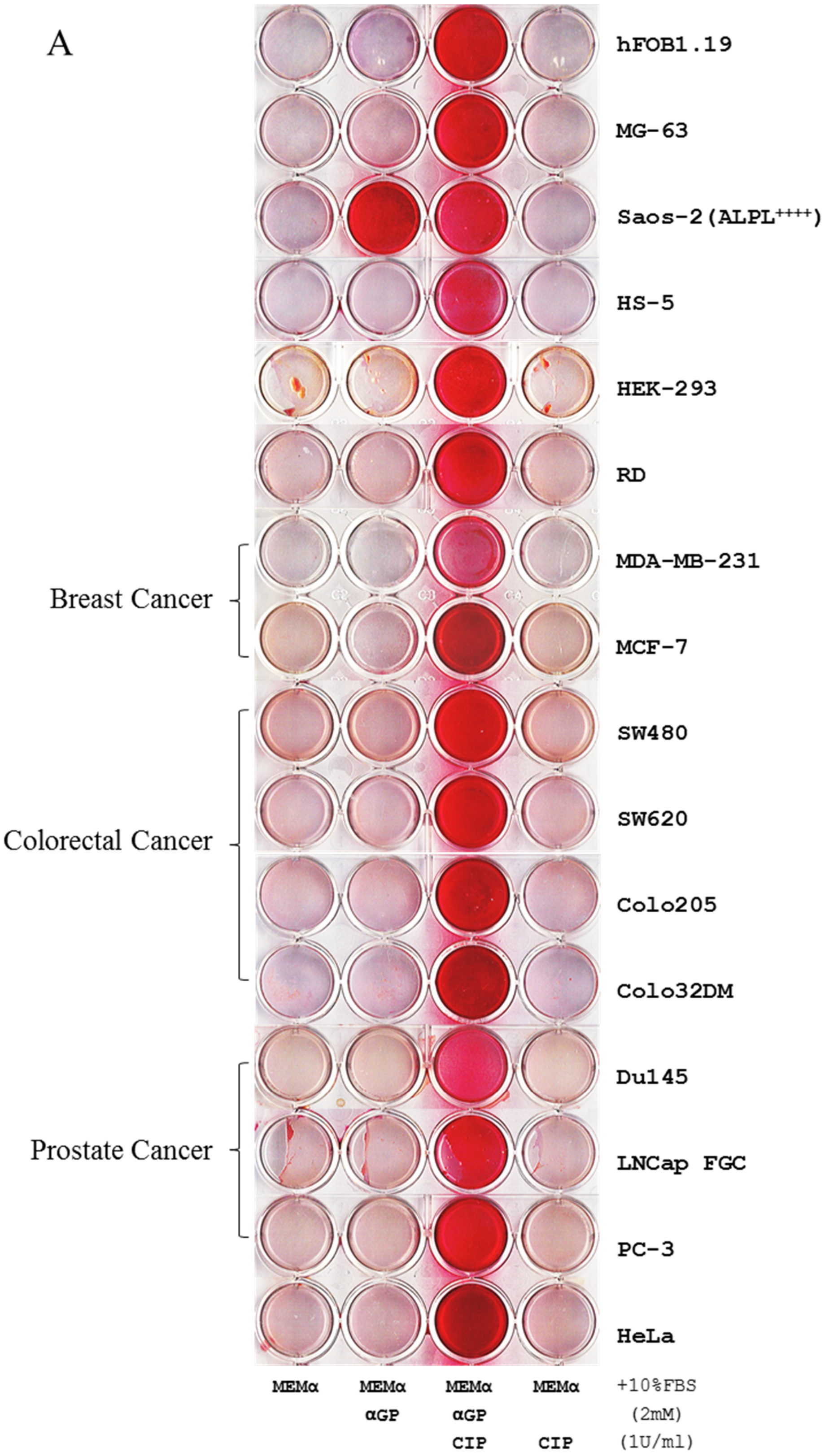
A
hFOB1.19
MG-63
Saos-2(ALPL++++)
HS-5
HEK-293
RD
Breast Cancer
MDA-MB-231
MCF-7
Colorectal Cancer
SW480
SW620
Colo205
Colo32DM
Prostate Cancer
Du145
LNCap FGC
PC-3
HeLa
MEMα
MEMα αGP
MEMα αGP CIP
MEMα CIP
+10%FBS
(2mM)
(1U/ml)

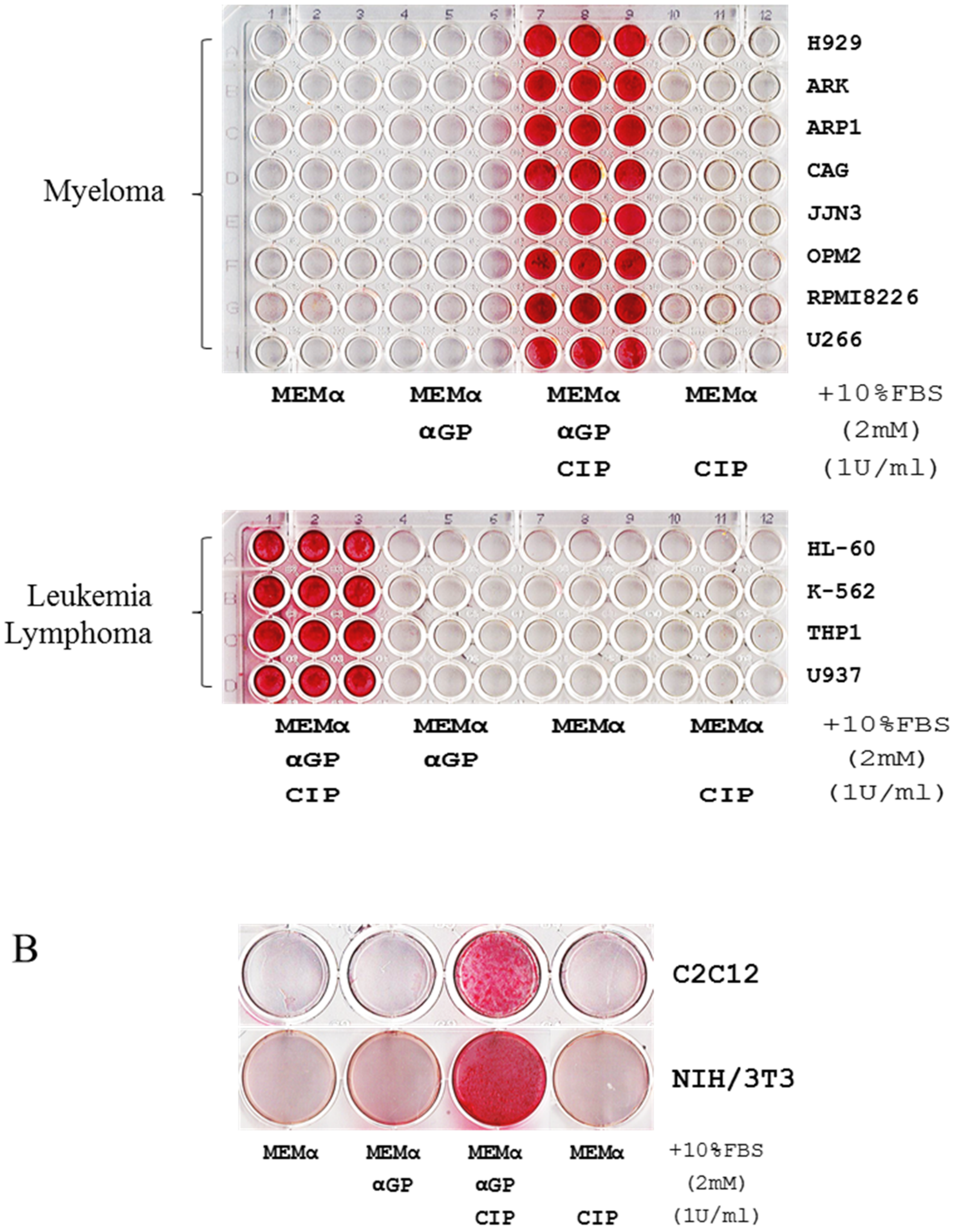

Figure. S3. Biomineralization of human and mouse cells without induction of cellular differentiation. Alizarin Red S staining revealed mineralization in (A) human cell lines (n=28) and (B) mouse cell lines (n=2) after 7 days of culture in MEM*α*/10% FBS supplemented with *α*GP (2 mM) and CIP (1 U/ml), the media were changed on day 4. The reaction did not occur if any one of the three elements was missing.

```
ALPL         1   MISPFLVLAIGTCLTNSLVPEKEKDPKYWRDQAQETLKYALELQKLNT  48
CIP          1 MQGACVLLLLGLHLQLSLGLVPVEEEDPAFWNRQAAQALDVAKKLQPIQT  50
SAP          1   MIGRTTFIALFVKVLTIWSFTKGED-CVWDNDVDYP-----EYPPLIL  42
                        . .   .          *   *                .

ALPL        49 NVAKNVIMFLGDGMGVSTVTAARILKGQLHHNPGEETRLEMDKFPFVALS  98
CIP         51 -AAKNVILFLGDGMGVPTVTATRILKGQMNGKLGPETPLAMDQFPYVALS  99
SAP         43 DSSFQLVLPVLEGD-------QRITSVQS----GSELILACPGREISALG  81
                 . .... . .*         **   *     * *  *        **

ALPL        99 KTYNTNAQVPDSAGTATAYLCGVKANEGTVGVSAATERSRCNTTQGNEVT 148
CIP        100 KTYNVDRQVPDSAGTATAYLCGVKGNYRTIGVSAAARYNQCKTTRGNEVT 149
SAP         82 ---SEDAQATCLGG----KLVEVDGKEWNIVELGCTKMASETIHR----- 119
                      *     *     *  *     ..   ..       .

ALPL       149 SILRWAKDAGKSVGIVTTTRVNHATPSAAYAHSADRDWYSDNEMPPEALS 198
CIP        150 SVMNRAKKAGKSVGVVTTTRVQHASPAGAYAHTVNRNWYSDADLPADAQM 199
SAP        120 -NLGQCGDQ--DLGIYEVIGFDLPTTGHFY-----------------ELI 149
                 .  .      .*.         .    *

ALPL       199 QGCKDIAYQLMHNIRDIDVIMGGGRKYMYPKNKTDVEYESDEKARGTRLD 248
CIP        200 NGCQDIAAQLVNNM-DIDVILGGGRKYMFPVGTPDPEYPDDASVNGVRKR 248
SAP        150 RVCFDPANETTIFS--ENIVHGAS----IAAKDIDPGRPSFKTSTGFFSV 193
                 * * * .         .. *            *          *

ALPL       249 GLDLVDTWKSFKPRYKHSHFIWNRTELLTLDPHN-VDYLLGLFEPGDMQY 297
CIP        249 KQNLVQAWQ---AKHQGAQYVWNRTALLQAADDSSVTHLMGLFEPADMKY 295
SAP        194 SMISVYSQR-------------NQLELMKNLLGD-DELAATIIDPSKQFY 229
                   * . .             *.  *.             . .*    *

ALPL       298 ELNRNNVTDPSLSEMVVVAIQILRKNPKGFFLLVEGGRIDHGHHEGKAKQ 347
CIP        296 NVQQDHTKDPTLQEMTEVALRVVSRNPRGFYLFVEGGRIDHGHHDDKAYM 345
SAP        230 FAKGHMAPDADFVTVVEQDATYYYINALPQWQAFNNG---NWKYLEYDTR 276
                       *     .          *         .*   . .

ALPL       348 ALHEAVEMDRAIGQAGSLTSSEDTLTVVTADHSHVFTFGGYTPRGNSIFG 397
CIP        346 ALTEAGMFDNAIAKANELTSELDTLILVTADHSHVFSFGGYTLRGTSIFG 395
SAP        277 DLAEKHGTDLTVYSGG-----WGVLELEDINGNPVEIYLGLAQDKK---- 317
                * *    * ..   .        * .       *  . * .

ALPL       398 LAPMLSDTDKKPFTAILYGNGPGYKVVGGERENVSMVDYAHNNYQAQSAV 447
CIP        396 LAPSKALDSKS-YTSILYGNGPGYALGGGSRPDVNDSTSEDPSYQQQAAV 444
SAP        318 VVPAPALTWKVIYEKDTN---RAAAIVGINNPHITTAP-----------E 353
               . *  .   *  .            . *     .

ALPL       448 PLRHETHGGEDVAVFSKGPMAHLLHGVHEQNYVPHVMAYAACIGANLGHC 497
CIP        445 PQASETHGGEDVAVFARGPQAHLVHGVEEETFVAHIMAFAGCVEPYTDCN 494
SAP        354 PLCTDICSSLTWLDFDFG---DLVHGYTYCCSVADLRAAIPNVPDLGDVD 400
               *   .         *  *    *.**      *  . *    .

ALPL       498 APASS-------AGSLAAGPLLLALALYPLSVLF*     525
CIP        495 LPAPTTATSIPDAAHLAASPPPLALLAGAMLLLLAPTLY 533
SAP        401 ILDE                                    404
```

Figure. S4. Alignment of amino acid sequences of human tissue-nonspecific ALP (ALPL), calf intestinal ALP (CIP), and shrimp hepatopancreas ALP (SAP). ALPs from three distant species across Kingdom Animalia demonstrate substantial differences; 48 residues are conserved (*).
